# Comparison of Clinical Outcomes Between Femtosecond Laser-assisted Cataract Surgery and Phacoemulsification in Patients with Diabetes: A Systematic Review

**DOI:** 10.18502/jovr.v21.18209

**Published:** 2026-07-15

**Authors:** Luksanaporn Krungkraipetch, Nattawat Asawaworarit, Rosepon Asawaworarit, Pakaphan Dinchuthai

**Affiliations:** ^1^Department of Ophthalmology, Faculty of Medicine, Burapha University, Chonburi, Thailand; ^2^Department of Internal Medicine, Faculty of Medicine, Burapha University, Chonburi, Thailand

**Keywords:** Corneal Thickness, Diabetes Mellitus, Endothelial Cell Density, Femtosecond Laser-assisted Cataract Surgery, Phacoemulsification, Systematic Review

## Abstract

This article systematically review and compare the efficacy and safety of femtosecond laser-assisted cataract surgery (FLACS) and traditional phacoemulsification (PE) in patients with diabetes. A systematic search was conducted in Scopus, PubMed, Cochrane Library, and Google Scholar from inception to December 2024. Studies comparing FLACS with PE in patients with diabetes were included. The primary outcomes were central corneal thickness (CCT) and endothelial cell density (ECD), while the secondary outcomes included effective phaco time (EPT), surgical complications, and visual outcomes. The Cochrane Risk of Bias Tool 2.0 was used to assess quality in randomized controlled trials (RCTs), ROBINS-I for cohort studies, and the Newcastle-Ottawa scale for retrospective studies. Six studies involving 345 eyes met the inclusion criteria: one RCT, two cohort studies, one prospective study, and two retrospective studies. In the RCT, FLACS demonstrated significantly higher postoperative ECD compared to PE (mean difference: 423.55 cells/mm²; 95% CI: 199.89 to 647.21; *P* < 0.001). One cohort study reported EPT values of 6.52 ± 4.93 seconds for FLACS and 5.63 ± 4.31 seconds for PE. No significant differences in CCT were observed between groups at 3 months postoperatively across multiple studies. One study reported a lower incidence of postoperative complications, including fibrin exudation, corneal edema, posterior synechiae, and pigment dispersion, in the FLACS group, although specific rates were not provided. Overall, FLACS demonstrated superior endothelial cell preservation and a favorable safety profile in patients with diabetes; however, the available evidence is limited by study heterogeneity and risk of bias. Large-scale randomized controlled trials with standardized outcome reporting are needed.

##  INTRODUCTION

Cataract surgery is among the most often performed operations in ophthalmology, with phacoemulsification (PE) traditionally regarded as the gold standard.^[1]^ Recently, femtosecond laser-assisted cataract surgery (FLACS) has emerged as a viable option, promising increased precision and potentially better surgical outcomes. FLACS introduces automation to key aspects of the procedure, including corneal incision, capsulotomy, and lens fragmentation, which may improve both safety and efficacy.^[12]^

In patients with diabetes, cataract surgery poses challenges due to the presence of associated ocular comorbidities. These individuals are at greater risk for postoperative complications, including macular edema, delayed wound healing, and decreased endothelial cell density (ECD). ${}^{\text{[34]}}$ Such factors can adversely affect surgical outcomes, making the choice of technique critically important in this population. Moreover, diabetes can lead to alterations in corneal thickness and endothelial health, resulting in less predictable recovery compared to nondiabetic individuals.^[3,4,5]^

Given the increasing use of both FLACS and PE in cataract surgery, it is crucial to determine whether the advanced capabilities of FLACS translate into significantly better clinical outcomes for this group of patients. Numerous studies have explored the comparative outcomes of FLACS and PE in this demographic, focusing on parameters such as best-corrected visual acuity (BCVA), central corneal thickness (CCT), ECD, and postoperative complications like cystoid macular edema (CME).^[6,7,8,9,10]^ However, literature has not agreed on whether one technique is superior to the other in patients with diabetes. Some studies suggest that FLACS may offer benefits, including faster recovery, reduced surgical energy, and minimized corneal damage. In contrast, others find no significant difference between FLACS and PE in terms of visual outcomes or corneal health after surgery. The diversity in study designs, patient populations, and follow-up durations has made it challenging to draw definitive conclusions.^[11,12,13]^

The purpose of this systematic review is to synthesize current evidence to determine whether FLACS offers measurable clinical advantages over PE in patients with diabetes, thereby informing surgical decision-making in this high-risk group.

##  METHODS

### Study Protocol and Registration

**Figure 1:**
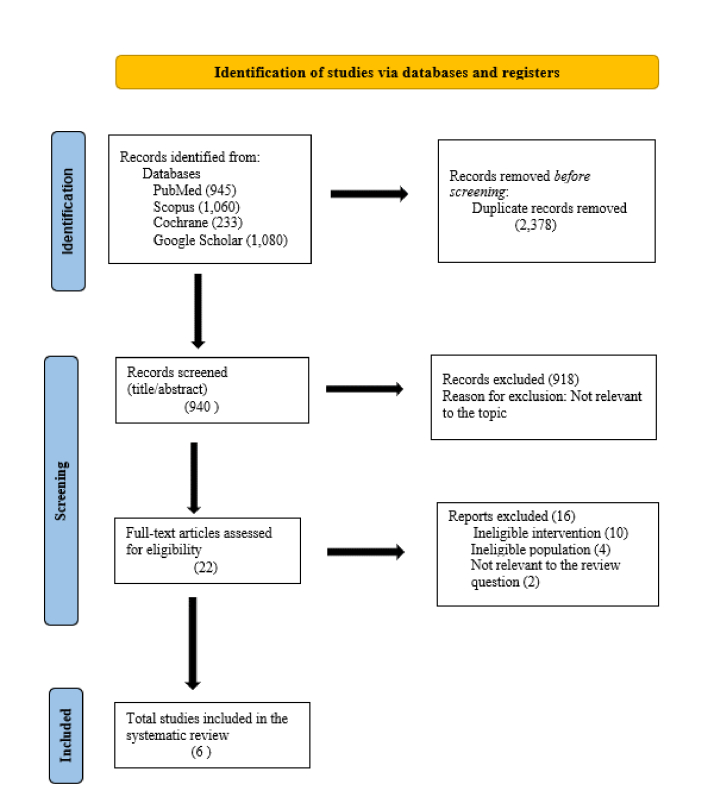
PRISMA flow diagram.

We thoroughly evaluated the Preferred Reporting Items for Systematic Reviews and Meta-Analyses (PRISMA) criteria, as described in the Supplementary Material. The study protocol was registered with the International Prospective Register of Systematic Reviews (PROSPERO). The CRD for Prospero registration is CRD 42024559462. A completed PRISMA 2020 checklist^[14]^ is provided in Supplementary Material 1. Figure 1 shows a PRISMA flow diagram describing the study selection process, including reasons for exclusion.

### Eligibility Criteria 

To assess any intervention aimed at improving the existing state of FLACS. These interventions involved empirical research studies, a global geography context, and years of analysis, and were published in English between 2000 and 2024.

### Source Strategies and Data Search 

In January 2025, we did a comprehensive search of PubMed, Cochrane CENTRAL, Google Scholar, and Scopus databases for relevant studies published between 2000 and 2024. The method followed the PICO (Participants, Intervention, Comparators, Outcome) framework and was implemented by two investigators. Keywords and medical terminology were obtained from PubMed and the Cochrane Library MeSH. EndNote Version 20 software was used to manage duplicate records, with a step-by-step syntax, as described in Supplementary Material 2. Full Boolean search strings, including MeSH terms and operators, are presented in Supplementary Material 2 (For example, “Femtosecond Laser-Assisted Cataract Surgery”[MeSH] OR “FLACS” AND “Phacoemulsification”[MeSH] OR “PE” AND “Diabetes Mellitus”). Gray literature, such as conference abstracts and theses, was excluded. Only English-language publications were included.

### Study Selection

Discrepancies in screening and data extraction were handled through consensus. If consensus was not obtained, a third reviewer was consulted.

### Inclusion criteria

• Study types: randomized controlled trials (RCTs), prospective cohort studies, retrospective comparative studies, case–control studies
• Population: adult patients ( ≥ 18 years) diagnosed with diabetes mellitus requiring cataract surgery
• Interventions: FLACS vs conventional PE, clear reporting of surgical technique
• Outcomes: Non-comparative studies, defined as those not directly comparing FLACS and PE studies with unclear outcome measures, namely, those that lacked clearly defined primary or secondary outcome metrics or statistical reporting (e.g., no standard deviation or effect size)
  ∘ Primary: ECD, CCT
  ∘ Secondary: BCVA, effective phaco time (EPT), surgical complications, recovery time

#### Exclusion criteria 

Case reports/series, non-comparative studies, studies without clear outcome measures, duplicate publications, and conference abstracts without full data.

### Risk of Bias Assessment 

The designated data points of each study were collected using a standardized data extraction form. Two independent reviewers collected qualitative and quantitative data and assessed the risk of bias for each of the included studies. Risk of bias in RCTs was assessed using the Cochrane Risk of Bias 2.0 tool (ROB 2).^[15]^ This tool evaluates five domains: deviations from intended interventions, measurement of outcomes, randomization process, missing outcome data, and selection of the reported results. For non-randomized cohort studies, the ROBINS-I (Risk of Bias in Non-randomized Studies of Interventions)^[16]^ tool was used to assess bias across seven domains: selection of participants, confounding factors, classification of interventions, missing data, measurement of outcomes, deviations from intended interventions, and selection of the reported results. The risk bias in retrospective studies was assessed using the Newcastle-Ottawa scale,^[17]^ which evaluates study quality across three domains: selection of participants, comparability of groups, and ascertainment of outcomes.

Discrepancies in judgments (*n* = 138) were handled by consulting with a third reviewer.

### Data Extraction 

Data extraction was carried out using a standardized data extraction form. Each included study provided relevant information, such as the author(s), year of publication, study design, number of participants, and major conclusions. All database citations were imported into EndNote version 20, and the title, author, journal, and year of publication were assessed to identify any statistical duplication. One author manually removed the duplicates identified by EndNote. A data extraction form was built to extract predetermined data points from each study. Using full-text review, two reviewers separately extracted qualitative and quantitative data from each reference, including study design, number and type of participants, study duration, objectives, measurement, outcomes, and overall study quality. Missing outcomes were estimated using a standard deviation from other sources.

### Data Synthesis and Statistical Analysis

The purpose of synthesizing data from the reviewed studies is to compare the clinical outcomes of FLACS and PE in patients with diabetes. This was conducted using R version 4.4-1, with a focus on key metrics such as BCVA, ECD, CCT, central macular thickness (CMT), and CME.^[18]^ Qualitative synthesis comprised:

• Narrative summary of findings
• Subgroup analyses when possible
• Assessment of clinical heterogeneity
• Evaluation of consistency across studies

Statistical analysis included descriptive statistics, and heterogeneity was quantified using the I² statistic. Because of substantial heterogeneity (I² > 60%), a formal meta-analysis was not performed. Where applicable, subgroup and sensitivity analyses were considered. Vote-counting and effect-direction plots were used to provide visual summaries when meta-analysis was not feasible. Graphical tools and trend analyses were also applied where appropriate to present and interpret the findings.

##  RESULTS

### Study Selection and Characteristics

This systematic review focused on six studies^[19,20,21,22,23,24]^ that met the inclusion criteria: one RCT, two cohort studies, and three retrospective or prospective analyses. These studies evaluated key postoperative outcomes following FLACS or PE, including BCVA, ECD, CCT, CMT, and CME [Figure 1].

**Table 1:** Characteristics of included studies

**Study**	**Year**	**Country**	**Study design**	**Participants (No.)**	**Outcome measure**	**Follow-up duration**
Cruz et al^[19]^	2023	Brazil	RCT	DM with FLACS (48) DM with PE (47)	ECD, CCT	3 months
Ma et al^[20]^	2023	China	Cohort	DM with FLACS (54) DM with PE (53)	EPT, CDE, CECC, BCVA	1 month
Yang et al^[21]^	2024	China	Cohort	DM with FLACS (60) DM with PE (101)	EPT, CDE, ECD, CCT, CV, HEX	1 week, 1 and 3 months
Ma et al^[22]^	2021	China	Prospective	DM with FLACS (44) DM with PE (47)	CMT, SFCT, BCVA	1 week, 1, 3, 6, and 12 months
Kang et al^[23]^	2021	Korea	Retrospective	DM with FLACS (31) NonDM with FLACS (44)	ECD, CCT, CV, CME, BCVA	6 months
Tang et al^[24]^	2024	China	Retrospective	DM with FLACS (60) DM with PE (60)	BCVA, Astigmatism	1 week, 1 and 3 months
BCVA, best-corrected visual acuity; CCT, central corneal thickness; CDE, cumulative dissipated energy; CECC, corneal endothelial cell count; CME, cystoid macular edema; CMT, central macular thickness; CV, coefficient of variation in cell size; DM, diabetes mellitus; ECD, endothelial cell density; EPT, effective phaco time; FLACS, femtosecond laser-assisted cataract surgery; HEX, hexagonal cells; PE, phacoemulsification; SFCT, subfoveal choroidal thickness.						

**Table 2:** Risk of bias and study quality assessment

**Study (Year)**	**Study design**	**Sample size (** ***n*** **)**	**Follow-up**	**Quality assessment tool**	**Risk of bias**
Cruz et al (2023)^[19]^	RCT	FLACS (48) PE (47)	3 months	Cochrane RoB 2.0	Some concerns
Ma et al (2023)^[20]^	Cohort	FLACS (54) PE (53)	1 month	ROBINS-I	Moderate risk
Yang et al (2024)^[21]^	Cohort	FLACS (60) PE (101)	3 months	ROBINS-I	Low risk
Ma et al (2021)^[22]^	Prospective	FLACS (44) PE (47)	12 months	ROBINS-I	Low risk
Kang et al (2021)^[23]^	Retrospective	FLACS (31) NonDM (44)	6 months	NOS	7/9 stars
Tang et al (2024)^[24]^	Retrospective	FLACS (60) PE (60)	3 months	NOS	6/9 stars
FLACS, femtosecond laser-assisted cataract surgery; PE, phacoemulsification; DM, diabetes mellitus; RCT, randomized control trial; ROB, revised Cochrane risk-of-bias tool for randomized trials; NOS, Newcastle–Ottawa scale.					

The studies conducted in Brazil, China, and South Korea enrolled a total of 345 eyes, and the mean age of patients ranged from 57.1 to 58.7 years. Most participants (56-64%) were male, with diabetes duration ranging from 5 to 15 years and HbA1c values between 6.5% and 8.5%.

Study designs, population characteristics, and outcome measures are detailed in Table 1.

Table 1 summarizes the key characteristics of all included studies, including study design, country of origin, number of participants, intervention groups (FLACS vs PE), outcome measures, and follow-up duration. It provides context for interpreting the comparative outcomes across various study types and populations.

### Risk of Bias 

The methodological quality of included studies was evaluated using methods suited to each study design: the Cochrane Risk of Bias 2.0 tool for RCTs, the ROBINS-I tools for cohort studies, and the Newcastle-Ottawa scale for retrospective studies.

The included RCT by Cruz et al^[19]^ was graded as having “some concerns” due to unclear risk in certain domains. Cohort studies (Ma et al^[20]^ and Yang et al^[21]^) were assessed to have a moderate probability of bias, while the study by Ma et al^[22]^ was graded as low risk. Retrospective studies (Kang et al^[23]^ and Tang et al^[24]^) received Newcastle-Ottawa scores of 7/9 and 6/9, respectively. A summary of all bias assessments is presented in Table 2.

This table presents a structured evaluation of each study's risk of bias based on study design using appropriate tools. RCTs were assessed using the Cochrane RoB 2.0 tool, cohort studies with the ROBINS-I tool, and retrospective studies using the Newcastle-Ottawa scale. The ratings reflect the methodological rigor and potential limitations in each study.

### Primary Outcomes

#### Corneal endothelial cell outcomes

The forest plot showed the results for both ECD and CCT. The meta-analysis results provide valuable insights into how FLACS compares to PE in patients with diabetes. The pooled effect size for both ECD and CCT does not cross zero, suggesting that the comparison between FLACS and PE reveals a statistically significant difference. FLACS is significantly better at preserving endothelial cells (*P* < 0.001).However, the mean difference of 423.55 cells/mm², while statistically significant, may have limited clinical relevance, depending on baseline endothelial status and surgical risk [Table 3].

Table 3 compares two corneal health outcomes, ECD and CCT, between FLACS and PE. It includes numerical findings and *P*-values to highlight whether differences are statistically and/or clinically significant.

**Table 3:** Corneal endothelial and thickness outcomes

**Author**	**Outcome measure**	**Finding for FLACS**	**Finding for PE**	**Significance/Key results**
Cruz et al^[19]^	ECD	The mean ECD was 423.55 cells higher than PE (*P * < 0.001).	Lower ECD compared to FLACS.	FLACS was significantly better at preserving endothelial cells (*P * < 0.001).
Yang et al^[21]^	ECD	ECD was reduced in both groups: there were no differences between A1 and B1, but the A2 and A3 groups showed better PE results.	Lower ECD reductions in A2 and A3 groups (*P * < 0.05).	PE showed slightly better results for early postoperative ECD, though differences were transient.
Kang et al^[23]^	ECD	No significant differences between diabetic and nondiabetic groups.	Similar findings; no significant differences.	Diabetes did not significantly affect ECD outcomes for either type of surgery.
Cruz et al^[19]^	CCT	There were no significant differences after 3 months.	No significant differences after 3 months.	There were no long-term differences in CCT between FLACS and PE.
Yang et al^[21]^	CCT	Higher CCT at day 1 and week 1 post-op compared to PE.	Lower CCT early post-op; a transient benefit over FLACS. No significant differences after 3 months.	PE showed better early CCT recovery, but no long-term advantage over FLACS.
Kang et al^[23]^	CCT	Higher CCT in patients with diabetes at 1 month, but no differences at 3 months.	Higher CCT in patients with diabetes at 1 month, but no differences at 3 months.	Diabetes contributed to early post-op CCT increase, but no significant long-term differences.
FLACS, femtosecond laser-assisted cataract surgery; PE, phacoemulsification; ECD, endothelial cell density; CCT, central corneal thickness.				

#### Macular outcomes

Ma et al demonstrated that while both FLACS and PE groups experienced an increase in CMT 1 week after surgery, the FLACS group showed faster recovery, with CMT returning to baseline by month 3; the PE group did not return to baseline until month 12.^[21]^ This suggests that FLACS may lead to quicker resolution of macular edema. Tang et al further confirmed improved macular outcomes in the FLACS group, with better visual recovery and astigmatism correction after 6 months compared to the PE group [Table 4].^[25]^

Table 4 outlines results regarding CMT recovery and CME incidence. It emphasizes postoperative differences between FLACS and PE in macular recovery rates and incidence of inflammation-mediated complications in patients with diabetes.

**Table 4:** Central macular thickness and cystoid macular edema outcome

**Study**	**Outcome measure**	**Finding for FLACS**	**Finding for PE**	**Significance/Key results**
Ma et al^[20]^	CMT	CMT returned to baseline by month 3.	CMT returned to baseline by month 12.	FLACS led to faster recovery of CMT (month 3 vs month 12 for PE).
Kang et al^[23]^	CME	There was no incidence of CME in the study.	There was no incidence of CME in the study.	CME risk was higher in patients with diabetic retinopathy, but there was no clear difference between FLACS and PE in preventing CME.
FLACS, femtosecond laser-assisted cataract surgery; PE, phacoemulsification; ECD, endothelial cell density; CMT, central macular thickness; CME, cystoid macular edema.				

#### Best-corrected visual acuity (BCVA) outcome

Ma et al^[20]^ reported that BCVA was significantly better in the FLACS group postoperatively, indicating faster visual recovery than PE. Additionally, visual quality was higher in the FLACS group. Tang et al^[24]^ found that the FLACS group showed better improvements in naked-eye vision and a reduction in astigmatism at month 6 after surgery, with no significant differences noted in a forest plot. FLACS leads to faster and better visual recovery. Nevertheless, the magnitude of BCVA improvement, although statistically significant in some studies, have not always reached clinically meaningful thresholds, as shown in Table 5.

Table 5 summarizes two visual function outcomes: BCVA and astigmatism. It demonstrates whether FLACS leads to more rapid or sustained improvements in visual performance compared to conventional PE.

**Table 5:** Best-corrected visual acuity and astigmatism outcome.

**Study**	**Outcome measure**	**FLACS**	**PE**	**Significance/Key results**
Ma et al^[20]^	BCVA	Significantly better BCVA and visual quality 1 month after surgery compared to PE.	Lower BCVA and slower recovery compared to FLACS.	FLACS led to faster and better visual recovery.
Tang et al^[24]^	BCVA	Improved naked-eye vision and astigmatism 6 months after surgery.	Slower recovery of vision compared to FLACS.	FLACS offered better long-term visual outcomes after 6 months.
FLACS, femtosecond laser-assisted cataract surgery; PE, phacoemulsification; BCVA, best-corrected visual acuity.				

##  DISCUSSION

### Summary of Main Findings

This systematic review synthesizes the current evidence comparing FLACS and traditional PE in patients with diabetes. Our analysis suggests that FLACS may provide certain advantages, including reduced ultrasound energy use, better endothelial cell preservation, and potentially faster early visual recovery.^[25]^ However, the therapeutic relevance of these advantages is unknown, and the current evidence is limited.

### Comparison with Previous Literature

Prior systematic reviews in general cataract populations have reported mixed outcomes. Chen et al^[26]^ demonstrated comparable visual results but lower effective phacoemulsification time with FLACS, whereas Wang et al^[27]^ observed a reduced risk of posterior capsule rupture. In the diabetic subgroup, Johnson et al^[28]^ and Zhang et al^[29]^ reported better outcomes with FLACS, including reduced corneal trauma and faster recovery. However, none of these reviews focused exclusively on diabetic cohorts. Our review addresses this gap by providing diabetes-specific insights into outcomes such as endothelial cell loss, macular edema, and visual recovery.

### Interpretation of Findings

The observed benefit of FLACS in ECD preservation is consistent with the theoretical advantage of reduced ultrasound energy and surgical trauma.^[17,18]^ Nevertheless, the absolute difference ( ∼ 423 cells/mm²) may have limited clinical relevance unless baseline ECD is already compromised, such as in cases with long-standing diabetes or advanced cataracts.^[30]^

In contrast to nondiabetic cohorts, where FLACS and PE yield largely comparable visual outcomes,^[26,27,28]^ diabetic-specific factors such as impaired endothelial function, delayed wound healing, and increased susceptibility to macular edema may amplify differences between the two techniques.^[31]^ These factors highlight the need for stratified analyses and underscore the potential importance of surgical precision in this high-risk population.

### Limitations

Despite these promising findings, important limitations must be acknowledged. The current evidence base consists of a single RCT and several retrospective studies, most with short follow-up durations (1-3 months). Such limitations preclude strong conclusions about long-term outcomes, including CME, endothelial decompensation, or sustained visual benefit.^[32]^ In addition, potential complications associated with FLACS, such as capsular block syndrome, device-related issues, and cost burden, remain underreported and require further investigation.^[33]^

### Clinical Implications

For clinical practice, FLACS may be particularly advantageous in selected patients with diabetes, including those with borderline endothelial counts or advanced cataracts.^[19]^ However, PE remains a valid and often preferable option in many cases, given its lower cost, shorter surgical time, and broader accessibility. Personalized decision-making, which considers patient characteristics, ocular risk factors, and institutional resources, should guide the choice of surgical technique.

### Future Directions

Future research should focus on well-designed, multicenter RCTs with longer follow-up periods ( ≥ 12 months) to clarify the long-term safety, efficacy, and cost-effectiveness of FLACS in patients with diabetes. Economic analyses will be especially important in resource-limited settings where the cost of FLACS may be prohibitive.

##  SUMMARY

This systematic review provides the first focused synthesis of FLACS outcomes in diabetic cohorts, suggesting superior endothelial cell preservation and reduced postoperative complications compared to PE. However, due to limitations such as short follow-up periods and inclusion of nonrandomized studies, these findings should be regarded with caution. Larger, high-quality RCTs are necessary to validate these results and determine the clinical and economic value of FLACS in this population.

##  Financial Support and Sponsorship

None.

##  Conflicts of Interest 

None.
